# Coupling Reactions of α-Bromocarboxylate with Non-Aromatic *N*-Heterocycles ‡

**DOI:** 10.3390/molecules14083019

**Published:** 2009-08-13

**Authors:** Katerina Brychtova, Barbora Slaba, Lukas Placek, Josef Jampilek, Ivan Raich, Jozef Csollei

**Affiliations:** 1 Department of Chemical Drugs, Faculty of Pharmacy, University of Veterinary and Pharmaceutical Sciences, Palackeho 1-3, 612 42 Brno, Czech Republic; E-mails: brychtovak@vfu.cz (K.B.), csolleij@vfu.cz (J.C.); 2 Zentiva k.s., U kabelovny 130, 102 37 Prague 10, Czech Republic; E-mails: barbora.slaba@zentiva.cz (B.S.), lukas.placek@zentiva.cz (L.P.); 3 Department of Chemistry of Natural Compounds, Faculty of Food and Biochemical Technology, Institute of Chemical Technology, Technicka 5, 166 28 Prague 6, Czech Republic; E-mail: ivan.raich@vscht.cz (I.R.)

**Keywords:** C-N nucleophilic coupling, *N*-heterocycles, ω-lactams, copper catalyst, *ab initio*/DFT calculations

## Abstract

The conditions for the C-N bond forming reaction (C-N coupling reaction) between α-bromocarboxylate and nitrogen-containing non-aromatic heterocyclic rings under heterogeneous copper(I) oxide catalysis are investigated in this paper. All the generated compounds were fully characterized by IR, NMR and MS spectroscopy. *Ab initio*/DFT calculations of partial charges on nitrogen atoms in all the discussed heterocycles and on C_(2)_ of carboxylate under applied conditions were predicted. These *in silico* results correlate relatively with the experimental observations.

## 1. Introduction

Transdermal penetration enhancers (also called sorption promoters or accelerants) are special pharmaceutical excipients that interact with skin components to increase the penetration of drugs from topical dosage forms to blood circulation [1,2,3]. Numerous compounds (with different chemical structures) have been evaluated as penetration enhancers and a number of potential sites and modes of action were identified [1,3]. Some of the important penetration enhancers, as classified by Sinha and Kaur [4], are terpenes and terpenoids, pyrrolidinones, fatty acids and esters, sulfoxides, alcohols and glycerides and miscellaneous enhancers including phospholipids, cyclodextrin complexes, amino acid derivatives, lipid synthesis inhibitors, clofibric acid, dodecyl-*N*,*N-*dimethylamino acetate and enzymes.

As part of a project directed at the synthesis of new potential transdermal penetration enhancers based on the structure of 6-aminohexanoic acid derivatives [1,3], the problem of C-N coupling reactions of ethyl-2-bromo-6-(2,5-dioxopyrrolidin-1-yl)hexanoate (**2**) and several nitrogen-containing saturated rings, including mainly basic heterocycles, ω-lactams and a cyclic imide, was solved. Nitrogen-containing heterocycles are very important targets in the organic chemistry. They are abundant in natural products and in pharmaceutical agents. A number of various compounds containing C-N bond have important biological, pharmaceutical, or material properties [5,6,7].

Herein the utility of copper(I) oxide as a heterogeneous catalyst in the process of C-N bond forming reactions is reported. Several reviews describing recent progress of copper-mediated coupling reactions for C-N bond formation have been published [8,9,10] and nucleophilic substitutions using copper(I) catalysts were described in other papers where copper(I) oxide [11,12], sulfide [13,14], iodide [15,16] or other copper(I) derivatives were used [17]. Contrary to the above referred articles dealing mostly with copper-mediated arylation of aromatic or aliphatic amines, coupling of the aliphatic compounds is discussed in this paper.

## 2. Results and Discussion

**Scheme 1:**
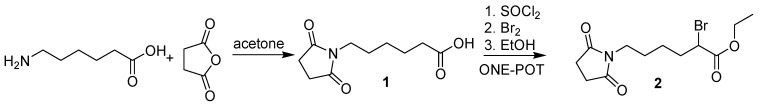
Synthesis of ethyl-2-bromo-6-(2,5-dioxopyrrolidin-1-yl)hexanoate (**2**).

The starting material ethyl-2-bromo-6-(2,5-dioxopyrrolidin-1-yl)hexanoate (**2**) was prepared by multistep synthesis from 6-aminohexanoic acid. This amino acid was condensed with succinic anhydride to obtain succinimide intermediate **1**, which was then transformed by means of one-pot synthesis under the optimized Schwenk and Papa procedure conditions [18,19] to α-bromocarboxylate **2**. The synthesis route is shown in Scheme 1. This synthesis was reported recently in [20], dealing with the problems associated with the generation of α-bromocarboxyl compounds and their reaction with pyrrolidin-2-one under different conditions and describing various synthetic by-products.

During the process of preparation of adducts with cyclic amines (compounds **3a-3c**) the coupling reaction of pyrrolidine, piperidine and morpholine with compound **2** was successful under conventional conditions (Method A) and provided very satisfactory yields (Table 1). The key interest was to prepare derivatives with ω-lactam substitution at the α position of the carboxylate, but the coupling reaction of compound **2** and the ω-lactam ring either did not occur under any conventional conditions (e.g. Method A) or undesirable products were obtained [20]. To overcome these difficulties, special conditions were used in Method B, in particular, a specific heterogeneous copper catalyst – powdered copper(I) oxide.

When piperidin-4-one was used as a positional isomer of the 6-membered ω-lactam ring for nucleophilic coupling under the conditions of Method A (compound **3g**), a yield comparable to that obtained with Method B was achieved. In the coupling reaction of pyrrolidin-2,5-dione and compound **2** Method A for the did not give any of compound **3h**, therefore Method B was used. It yielded 66% of **3h**. Attempts were made to prepare compounds **3d**-**3f**, **3h** under the conditions of Method B, but without copper heterogeneous catalyst. In all cases no product was obtained. Compounds **3a**-**3c** were additionally prepared under conditions of Method B. The yields and the used methods are summarized in Table 1.

These results were supported by *ab initio*/DFT calculations [21,22,23,24] of partial charges. All the calculated data are shown in Table 1. *Ab initio*/DFT calculations of partial charges on C_(2)_ of the carboxylate in Method A (in toluene) is –0.01 and in Method B (in DMF) is –0.30. It means, that C_(2)_ of the carboxylate possesses relatively negative charges under the conditions of both methods and it can be assumed that a nucleophilic substitution is not the preferred reaction, *i.e.* the C_(2)_ position is not activated for nucleophilic attack.

According to Table 1, it may be concluded that the calculated negative partial charge on the nitrogen atom of nucleophile in the range from –0.48 to –0.55 (ω-lactams and cyclic imide) is not sufficient for successful nucleophilic substitution. When the nucleophile possesses the computed value of charge –0.73, the coupling reaction is possible. This was conformed by very similar yields of compound **3g** generated using both Methods A and B. Taking into account the above mentioned facts, it can be assumed that the reaction mechanism could be a combination of S_N_1 and S_N_2 in Method A, or radical-ionic substitution using heterogeneous copper catalyst in Method B [13,14].

**Figure 1:**
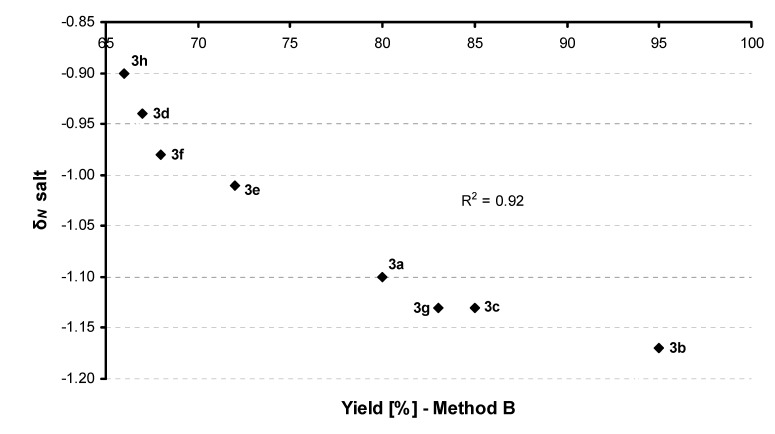
Correlation between experimental (Method B) and calculated data (δ*_N_* salt).

The interdependence between experimental yields of Method B and *ab initio*/DFT calculation data for δ*_N_* salt is illustrated in Figure 1. The deviation of this dependence is R^2^=0.92. The dependence deviation between experimental (yields of Method A) and calculation (δ*_N_* base) values for compounds **3a**-**3c** and **3g** is R^2^=0.67, but only for **3a**-**3c** the dependence deviation is 0.97. Piperidin-4-one (starting material for **3g**) as a position isomer of piperidin-2-one (starting material for **3e**) is a specific compound; it does not possess physico-chemical properties of 6-membered ω-lactam or cyclic 6-membered amine and therefore should not be included to this dependence. According to these deviations (R^2^=0.92, R^2^=0.97) it can be concluded that experimental and predicted data relatively correlate.

**Table 1:** The C-N coupling reactions of compound **2** with nitrogen-containing heterocycles and calculated partial charges on nitrogen atoms (δ*_N_*) of the free bases in toluene or the sodium salts in dry DMF.

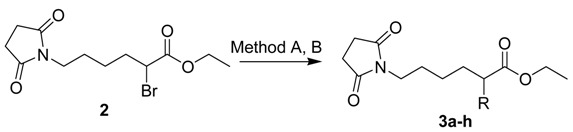					
**Comp.**	**R**	**Conditions*^a^***	**Yield (%)**	**δ*_N_* base**	**δ*_N_* salt**
**3a**	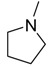	Method A	94	–0.77	–1.10
		Method B	80		
**3b**	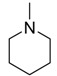	Method A	77	–0.73	–1.17
		Method B	95		
**3c**	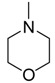	Method A	80	–0.73	–1.13
		Method B	85		
**3d**	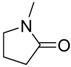	Method A	0	–0.50	–0.94
		Method B	67		
**3e**	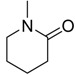	Method A	0	–0.55	–1.01
		Method B	72		
**3f**	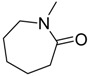	Method A	0	–0.53	–0.98
		Method B	68		
**3g**	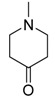	Method A	81	–0.76	–1.13
		Method B	83		
**3h**	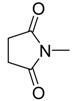	Method A	0	–0.48	–0.90
		Method B	66		

*^a^* Reaction conditions: Method A: toluene, reflux, 5h; Method B: NaH, DMF, Cu_2_O, reflux, 9h.

All eight compounds **3a**-**3h** prepared in this article are intermediates from which alkyl-6-(2,5-dioxopyrrolidin-1-yl)-2-(substituted)hexanoates with C_6_–C_12_ linear alkyl ester chains will be prepared. The preliminary results were presented recently [25]. The intermediates **3a**-**3h** do not meet the requirements/recommendations for effective transdermal penetration enhancers [1,3,4], in particularly they possess low hydrophobicity in comparison with substitution of ethyl esters by C_6_-C_12_ linear alkyl chains.

## 3. Conclusions

A series of eight substituted ethyl-6-(2,5-dioxopyrrolidin-1-yl)hexanoate derivatives and two intermediates were prepared from 6-aminohexanoic acid. Ten newly prepared compounds were characterized by ^1^H-, ^13^C-NMR spectra and IR spectra. Reaction conditions for the coupling of ethyl-2-bromo-6-(2,5-dioxopyrrolidin-1-yl)hexanoate (**2**) with nitrogen-containing heterocycles were described and a radical-ionic mechanism for the substitution, catalyzed by heterogeneous copper catalyst, was proposed. According to the above discussed facts, it could be concluded that the coupling of compound **2** and heterocycles α-substituted with keto moiety gave products only in presence of copper heterogeneous catalyst. *Ab initio*/DFT calculations of partial charges on nitrogen atoms in all the discussed heterocycles and on C_(2)_ of carboxylate under the applied conditions were predicted. These *in silico* results correlated relatively with the experimental observations.

## 4. Experimental

### 4.1. General

All reagents were purchased from Sigma-Aldrich (Schnelldorf, Germany) or Merck (Darmstadt, Germany). Kieselgel 60, 0.040-0.063 mm (Merck) was used for column chromatography. TLC experiments were performed on alumina-backed silica gel 40 F254 plates (Merck). The plates were illuminated under UV (254 nm) and evaluated in iodine vapour. The melting points were determined on a Boetius PHMK apparatus (Nagema, Germany) and are uncorrected. All ^1^H- and ^13^C-NMR spectra were recorded on a Bruker Avance-500 FT-NMR spectrometer (500 MHz for ^1^H and 125 MHz for ^13^C, Bruker Comp., Karlsruhe, Germany). Chemical shifts are reported in ppm (δ) to internal Si(CH_3_)_4_, when diffused easily exchangeable signals are omitted. Infrared (IR) spectra were recorded on a Smart MIRacle™ ATR ZnSe for Nicolet™ 6700 FT-IR Spectrometer (Nicolet - Thermo Scientific, U.S.A.). The spectra were obtained by accumulation of 256 scans with 2 cm^-1^ resolution in the 4,000-600 cm^‑1 ^region. Mass spectra were measured using the LTQ Orbitrap Hybrid Mass Spectrometer (Thermo Electron Corporation, U.S.A.) with direct injection into APCI source (400 °C) in the positive mode.

### 4.2. Synthesis

*6-(2,5-Dioxopyrrolidin-1-yl)hexanoic acid* (**1**): A solution of succinic anhydride (45.0 g, 450.0 mmol) in acetone (230 mL) was added dropwise to a suspension of 6-aminohexanoic acid (34.4 g, 262.0 mmol) in acetone (140 mL). The reaction mixture was stirred at room temperature for 24 hours after which it was filtered and the pure white crystalline product was washed with acetone. Yield 82%. Mp 100–102 °C; IR (cm^-1^) 3315, 2929, 1688, 1560, 1414, 1250, 1183; ^1^H-NMR (DMSO-*d_6_*), δ: 12.05 (s, 1H, OH), 3.02 (t, 2H, *J*=7.0 Hz, NCH_2_), 2.90 (s, 4H, OCCH_2_CH_2_CO), 2.41 (t, 2H, *J*=6.0 Hz, OOCCH_2_), 2.32–2.15 (m, 4H, CH_2_), 1.52–1.24 (m, 2H, CH_2_); ^13^C NMR (DMSO-*d_6_*), δ: 174.25, 173.66, 38.27, 33.52, 28.72, 28.62, 25.83, 24.11; HR-MS: for C_10_H_16_O_4_N [M+H]^+^ calculated 214.2378 m/z, found 214.2377 m/z.

*Ethyl-2-bromo-6-(2,5-dioxopyrrolidin-1-yl)hexanoate* (**2**): To the organic acid **1 **(45.8 g, 214.8 mmol), held at 30 °C, SOCl_2_ (29.4 g, 247.0 mmol, 17.9 mL) was slowly added dropwise and the mixture was stirred at 60–80 °C until the gas evolution essentially stopped. Br_2_ (36.1 g, 225.5 mmol, 11.6 mL) was added dropwise at 80 °C at approximately the same rate as Br_2_ was consumed. Stirring was continued for several hours until the evolution of HBr nearly stopped. Absolute EtOH (27 mL) was added slowly to the crude acid chloride at 20–30 °C. After stirring overnight, the mixture was evaporated until dry in a vacuum and the residue was dissolved in Et_2_O (50 mL). The solution was washed with diluted NaHSO_3_ and water, the organic layer was dried over anhydrous MgSO_4_, filtered and the organic solvent was removed under rotary evaporation. The crude product was purified by flash chromatography on silica gel, eluting with EtOAc/petroleum ether. Yield 81%, colourless oil; *R*_F_ 0.37 (EtOAc/petroleum ether 1:1); IR (cm^-1^) 2939, 1730, 1692, 1436, 1399, 1143, 818; ^1^H-NMR (CDCl_3_), δ: 4.16 (q, 2H, *J*=7.0 Hz, OCH_2_), 4.11 (t, 1H, *J*=7.3 Hz, BrCH), 3.44 (t, 2H, *J*=7.2 Hz, NCH_2_), 2.64 (s, 4H, OCCH_2_CH_2_CO), 1.99 (q, 2H, *J*=7.3 Hz, CHCH_2_), 1.54 (qi, 2H, *J*=7.0 Hz, CH_2_), 1.43–1.15 (m, 2H, CH_2_), 1.23 (t, 3H, *J*=7.1 Hz, CH_3_); ^13^C-NMR (CDCl_3_), δ: 176.94, 169.43, 61.84, 45.63, 38.23, 34.17, 28.08, 26.77, 24.43, 13.82. HR-MS: for C_12_H_19_O_4_NBr [M+H]^+^ calculated 320.0492 m/z, found 320.0491 m/z.

*Compounds* **3a-h***. General procedures*: *Method A*: The appropriate nitrogen compound (13.4 mmol) was dissolved in toluene (25 mL) and compound **2** (6.7 mmol) was added. The mixture was refluxed under argon for 5 hours. The solvent was evaporated and the rest was suspended in Et_2_O, solid was filtered off, washed with Et_2_O and the filtrate was concentrated under reduced pressure. Purification by flash chromatography on silica gel, eluting with EtOAc/petroleum ether + 1% TEA or CH_2_Cl_2_/MeOH. *Method B*: Nitrogen compound (10 mmol) was added slowly to a suspension of NaH (11 mmol, 60% dispersion in mineral oil) in dry DMF (25 mL). The mixture was stirred for a few minutes until the evolution of hydrogen gas stopped. Compound **2 **(6.7 mmol) and Cu_2_O (1.7 mmol, 25 mol %) were then added, and the mixture was refluxed under argon for 9 hours. The cooled mixture was poured onto ice, filtered through Celite and extracted with CHCl_3_. The combined organic extracts were washed with water, dried over anhydrous MgSO_4_, filtered and the organic solvent was removed under rotary evaporation. Purification by flash chromatography on silica gel, eluting with EtOAc/petroleum ether/TEA or CH_2_Cl_2_/MeOH.

*Ethyl-6-(2,5-dioxopyrrolidin-1-yl)-2-(pyrrolidin-1-yl)hexanoate* (**3a**): For the coupling of pyrrolidine and compound **2** both Method A and Method B conditions were used. Yield: 94% (Method A), 80% (Method B); a light yellow oil; R*_F_* 0.27 (CH_2_Cl_2_/MeOH 95:5); IR (cm^-1^) 2985, 2939, 2870, 1736, 1690, 1408, 1382, 1147; ^1^H-NMR (CDCl_3_), δ: 4.19 (q, *J=*7.2 Hz, 2H, OCH_2_), 3.49 (t, *J=*7.4 Hz, 2H, NCH_2_), 3.07 (t, *J=*7.1 Hz, 1H, CH), 2.70 (s, 4H, OCCH_2_CH_2_CO), 2.66–2.52 (m, 4H, NCH_2_), 1.82–1.41 (m, 10H, CH_2_), 1.29 (t, *J=*7.2 Hz, 3H, CH_3_); ^13^C-NMR (CDCl_3_), δ: 177.08, 172.89, 66.41, 60.21, 50.47, 38.57, 30.93, 28.12, 27.50, 23.47, 23.25, 14.36; HR-MS: for C_16_H_27_O_4_N_2_ [M+H]^+^ calculated 311.1965 m/z, found 311.1966 m/z.

*Ethyl-6-(2,5-dioxopyrrolidin-1-yl)-2-(piperidin-1-yl)hexanoate* (**3b**): For the coupling of piperidine and compound **2** the conditions of Method A and Method B were used. Yield: 77% (Method A), 95% (Method B); a light yellow oil; R*_F_* 0.48 (CH_2_Cl_2_/MeOH 95:5); IR (cm^-1^) 2934, 2856, 2809, 1698, 1400, 1146; ^1^H-NMR (CDCl_3_), δ: 4.17 (q, *J=*7.2 Hz, 2H, OCH_2_), 3.50 (t, *J=*7.4 Hz, 2H, NCH_2_), 3.08 (t, *J=*7.4 Hz, 1H, CH), 2.70 (s, 4H, OCCH_2_CH_2_CO), 2.62–2.40 (m, 4H, NCH_2_), 1.83–1.36 (m, 12H, CH_2_), 1.28 (t, *J=*7.2 Hz, 3H, CH_3_); ^13^C-NMR (CDCl_3_), δ: 177.09, 172.22, 67.97, 59.86, 50.69, 38.66, 28.79, 28.12, 27.42, 26.50, 24.65, 23.54, 14.51; HR-MS: for C_17_H_29_O_4_N_2_ [M+H]^+^ calculated 325.2122 m/z, found 325.2120 m/z.

*Ethyl-6-(2,5-dioxopyrrolidin-1-yl)-2-(morpholin-4-yl)hexanoate* (**3c**): For the coupling of morpholine and compound **2** the conditions of Method A and Method B were used. Yield: 80% (Method A), 85% (Method B); a yellow oil; R*_F_* 0.53 (CH_2_Cl_2_/MeOH 95:5); IR (cm^-1^) 2948, 2855, 1697, 1400, 1150, 1114; ^1^H-NMR (CDCl_3_), δ: 4.18 (q, *J=*7.1 Hz, 2H, OCH_2_), 3.74–3.63 (m, 4H, OCH_2_morph.), 3.50 (t,*J=*7.4 Hz, 2H, NCH_2_), 3.10 (t, *J=*7.4 Hz, 1H, CH), 2.71 (s, 4H, OCCH_2_CH_2_CO), 2.66–2.48 (m, 4H, NCH_2_morph.), 1.80–1.52 (m, 4H, CH_2_), 1.45–1.27 (m, 2H, CH_2_), 1.29 (t, *J=*7.1 Hz, 3H, CH_3_); ^13^C-NMR (CDCl_3_), δ: 177.07, 171.72, 67.54, 67.38, 60.19, 49.95, 38.57, 28.29, 28.13, 27.38, 23.32, 14.48; HR-MS: for C_16_H_27_O_5_N_2_ [M+H]^+^ calculated 327.1914 m/z, found 327.1915 m/z.

*Ethyl-6-(2,5-dioxopyrrolidin-1-yl)-2-(2-oxopyrrolidin-1-yl)hexanoate* (**3d**): For the coupling of pyrrolidin-2-one and compound **2** the conditions of Method A and Method B were used. Yield: 0% (Method A), 67% (Method B); a light yellow oil; R*_F_* 0.27 (EtOAc/petroleum ether 10:1 + 1% TEA); IR (cm^-1^) 2927, 1767, 1687, 1401, 1284, 1187, 1153, 1027; ^1^H-NMR (CDCl_3_), δ: 4.66 (dd, 1H, *J^1^*=5.0 Hz, *J^2^*=10.6 Hz, CH), 4.16 (q, 2H, *J*=7.1 Hz, OCH_2_), 3.50 (t, 2H, *J*=7.2 Hz, NCH_2_), 3.54–3.29 (m, 2H, CH_2_pyrr.), 2.70 (s, 4H, OCCH_2_CH_2_CO), 2.42 (t, 2H, *J*=8.0 Hz, CH_2_pyrr.), 2.17–1.95 (m, 2H, CH_2_pyrr. and 1H from CH_2_CH), 1.78–1.56 (m, 2H, CH_2_ and 1H from CH_2_CH), 1.34–1.28 (m, 2H, CH_2_), 1.26 (t, 3H, *J*=7.1 Hz, CH_3_); ^13^C-NMR (CDCl_3_), δ: 177.13, 175.78, 170.76, 61.12, 53.51, 43.53, 38.26, 30.73, 28.08, 27.03, 23.35, 18.21, 14.07; HR-MS: for C_16_H_25_O_5_N_2_ [M+H]^+^ calculated 325.1758 m/z, found 325.1757 m/z.

*Ethyl-6-(2,5-dioxopyrrolidin-1-yl)-2-(2-oxopiperidin-1-yl)hexanoate* (**3e**): For the coupling of piperidin-2-one and compound **2** the conditions of Method A and Method B were used. Yield: 0% (Method A), 72% (Method B): a light yellow oil; R*_F_* 0.27 (EtOAc/petroleum ether 10:1 + 1% TEA); IR (cm^-1^) 2941, 2868, 1698, 1637, 1401, 1347, 1178; ^1^H-NMR (CDCl_3_), δ: 5.14 (dd, *J=*10.3, 5.3 Hz, 1H, CH), 4.24–4.06 (m, 2H, OCH_2_), 3.50 (t, *J=*7.1 Hz, 2H, NCH_2_), 3.29–3.17 (m, 2H, NCH_2_pip.), 2.70 (s, 4H, OCCH_2_CH_2_CO), 2.48–2.42 (m, 2H, OCCH_2_pip.), 2.01–1.53 (m, 8H, CH_2_), 1.37–1.23 (m, 2H, CH_2_), 1.26 (t, *J=*7.2 Hz, 3H, CH_3_); ^13^C-NMR (CDCl_3_), δ: 177.13, 171.16, 170.47, 61.00, 55.72, 44.13, 38.42, 32.21, 28.13, 27.48, 27.22, 23.46, 23.12, 20.98, 14.15; HR-MS: for C_17_H_27_O_5_N_2_ [M+H]^+^ calculated 339.1914 m/z, found 339.1914 m/z.

*Ethyl-6-(2,5-dioxopyrrolidin-1-yl)-2-(2-oxoazepan-1-yl)hexanoate* (**3f**): For the coupling of azepan-2-one and compound **2** the conditions of Method A and Method B were used. Yield: 0% (Method A), 68% (Method B); a light yellow oil; R*_F_* 0.27 (AcOEt/petroleum ether + 1% TEA); IR (cm^-1^) 2937, 2863, 1695, 1674, 1401, 1156; ^1^H-NMR (CDCl_3_), δ: 5.10 (dd, *J^1^*=9.9 Hz, *J^2^*=5.1 Hz, 1H, CH), 4.33–4.00 (m, 2H, OCH_2_), 3.50 (t, *J=*7.2 Hz, 2H, NCH_2_), 3.41–3.15 (m, 2H, NCH_2_azep.), 2.71 (s, 4H, OCCH_2_CH_2_CO), 2.64–2.51 (m, 2H, OCCH_2_azep.), 2.05–1.90 (m, 2H, CH_2_), 1.85–1.45 (m, 8H, CH_2_), 1.39–1.27 (m, 2H, CH_2_), 1.26 (t, *J=*6.6 Hz, 3H, CH_3_); ^13^C-NMR (CDCl_3_), δ: 177.18, 176.22, 171.47, 60.97, 57.06, 46.19, 38.41, 37.28, 29.91, 28.63, 28.11, 27.29, 23.46, 23.25, 14.12; HR-MS: for C_18_H_29_O_5_N_2_ [M+H]^+^ calculated 353.2071 m/z, found 353.2071 m/z.

*Ethyl-6-(2,5-dioxopyrrolidin-1-yl)-2-(4-oxopiperidin-1-yl)hexanoate* (**3g**): For the coupling of piperidin-2-one and compound **2** the conditions of Method A and Method B were used. Yield: 81% (Method A); 83% (Method B); a light orange oil; R*_F_* 0.59 (CH_2_Cl_2_/MeOH 95:5); IR (cm^-1^) 2941, 2865, 2822, 1697, 1401, 1344, 1159; ^1^H-NMR (CDCl_3_), δ: 4.16 (q, *J=*7.1 Hz, 2H, OCH_2_), 3.52 (t, *J=*7.1 Hz, 2H, NCH_2_), 3.31 (t, *J=*7.5 Hz, 1H, CH), 3.04–2.76 (m, 4H, NCH_2_), 2.71 (s, 4H, OCCH_2_CH_2_CO), 2.51–2.34 (m, 4H, CH_2_COCH_2_), 1.80–1.33 (m, 6H, CH_2_), 1.28 (t, *J=*7.1 Hz, 3H, CH_3_); ^13^C-NMR (CDCl_3_), δ: 208.75, 177.09, 171.72, 66.51, 60.27, 48.97, 41.95, 38.51, 28.95, 28.11, 27.29, 23.50, 14.42; HR-MS: for C_17_H_27_O_5_N_2_ [M+H]^+^ calculated 339.1914 m/z, found 339.1915 m/z.

*Ethyl-2,6-bis(2,5-dioxopyrrolidin-1-yl)hexanoate* (**3h**): For the coupling of pyrrolidin-2,5-dione and compound **2** the conditions of Method A and Method B were used. Yield: 0% (Method A), 66% (Method B); a white crystalline compound; Mp 99–101 °C; R*_F_* 0.35 (EtOAc); IR (cm^-1^) 2980, 2940, 1690, 1391, 1253, 1190; ^1^H-NMR (CDCl_3_), δ: 4.62 (dd, *J^1^*=9.4 Hz, *J^2^*=5.8 Hz, 1H, CH), 4.19 (dq, *J^1^*=7.1 Hz, *J^2^*=1.6 Hz, 2H, OCH_2_), 3.47 (t, *J=*7.1 Hz, 2H), 2.79 (s, 4H, OCCH_2_CH_2_CO), 2.70 (s, 4H, OCCH_2_CH_2_CO), 2.18–2.05 (m, 2H, CH_2_), 1.64–1.46 (m, 2H, CH_2_), 1.30–1.22 (m, 2H, CH_2_), 1.24 (t, *J=*7.1 Hz, 3H, CH_3_); ^13^C-NMR (CDCl_3_), δ: 177.26, 176.54, 168.49, 61.80, 52.59, 38.33, 28.13, 28.08, 27.34, 26.97, 23.61, 14.04; HR-MS: for C_16_H_23_O_6_N_2_ [M+H]^+^ calculated 339.1551 m/z, found 339.1551 m/z.

### 4.3. Ab initio/DFT calculations

Geometry optimizations of all compounds were performed first at HF/6-31G(d,p) *ab initio* level in the gas phase and then reoptimized at B3LYP/6-31G(d,p) level in toluene or dimethylformamide. For nitrogen bases, both the free base and anion forms were taken into account. Solvents were simulated using the CPCM polarizable conductor calculation solvation model [21]. Charges for optimized structures were calculated at B3LYP/6-31G(d,p) level under the same solvent conditions using the Merz, Singh and Kollman procedure [22,23]. All *ab initio*/DFT calculations were performed in Gaussian 03W [24]. All the calculated data are shown in Table 1.

## References

[1] Williams A.C., Barry B.W. (2004). Penetration enhancers. Adv. Drug Deliv. Rev..

[2] Hrabalek A., Dolezal P., Farsa O., Sklubalova Z., Kunes J. (2000). Esters of 6-dimethylaminohexanoic acid as skin penetration enhancers. Pharmazie.

[3] Pugh W.J., Wong R., Falson F., Michniak B.B., Moss G.P. (2005). Discriminant analysis as a tool to identify compounds with potential as transdermal enhancers. J. Pharm. Pharmacol..

[4] Sinha V.R., Kaur M.P. (2000). Permeation enhancers for transdermal drug delivery. Drug. Dev. Ind. Pharm..

[5] Katritzky A.R., Rees C.W., Scriven E.F.V. (1996). Comprehensive Heterocyclic Chemistry II.

[6] Taylor J.B., Triggle D.J. (2007). Comprehensive Medicinal Chemistry II.

[7] Schreiber S.L. (2000). Target-oriented and diversity-oriented organic synthesis in drug discovery. Science.

[8] Evano G., Blanchard N., Toumi M. (2008). Copper-mediated coupling reactions and their applications in natural products and designed biomolecules synthesis. Chem. Rev..

[9] Xi Z., Liu F., Zhou Y., Chen W. (2008). CuI/L(L=pyridine-functionalized 1,3-diketones) catalyzed C–N coupling reactions of aryl halides with NH-containing heterocycles. Tetrahedron.

[10] Halfen J.A. (2005). Recent advances in metal-mediated carbon-nitrogen bond formation reactions. Aziridination and amidation. Curr. Org. Chem..

[11] Shen G., Lv X., Qian W., Bao W. (2008). Cu_2_O-catalyzed Ullmann-type reaction of vinyl bromides with imidazole and benzimidazole. Tetrahedron Lett..

[12] Huang Y.-Z., Miao H., Zhang Q.-H., Chen C., Xu J. (2008). Cu_2_O: a simple and efficient reusable catalyst for *N*-arylation of nitrogen-containing heterocycles with aryl halides. J. Catal. Lett..

[13] Jampilek J., Dolezal M., Kunes J., Raich I., Liska F. (2005). 4-Substituted aryl bromides coupling with 4-methoxybenzene-1-thiol by means of copper catalysts. Chem. Pap..

[14] Jampilek J., Dolezal M., Kunes J., Satinsky D., Raich I. (2005). Novel regioselective preparation of 5-chloropyrazine-2-carbonitrile from pyrazine-2-carboxamide and coupling study of substituted phenylsulfanylpyrazine-2-carboxylic acid derivatives. Curr. Org. Chem..

[15] Phillips D.P., Hudson A.R., Nguyen B., Lau T.L., McNeill M.H., Dalgard J.E., Chen J.-H., Penuliar R.J., Miller T.A., Zhi L. (2006). Copper-catalyzed *N*-arylation of oxindoles. Tetrahedron Lett..

[16] Filipski K.J., Kohrt J.T., Casimiro-Garcia A., Van Huis C.A., Dudley D.A., Cody W.L., Bigge C.F., Desiraju S., Sun S., Maiti S.N., Jaber M.R., Edmunds J.J. (2006). A versatile copper-catalyzed coupling reaction of pyridin-2(1*H*)-ones with aryl halides. Tetrahedron Lett..

[17] Kuil M., Bekedam E.K., Visser G.M., van den Hoogenband A., Terpstra J.W., Kamer P.C.J., van Leeuwen P.W.N.M., van Strijdonck G.P.F. (2005). Mild copper-catalyzed *N*-arylation of azaheterocycles with aryl halides. Tetrahedron Lett..

[18] Schwenk E., Papa D. (1948). Bromination of dicarboxylic acids. J. Am. Chem. Soc..

[19] Berry J.P., Isbell A.F., Hunt G.E. (1972). Amino phosphonic acids. II. Aminoalkylphosphonic acids. J. Org. Chem..

[20] Brychtova K., Farsa O., Csollei J. (2009). Copper catalyzed coupling of α-bromocarboxylate with ω-lactam. Lett. Org. Chem..

[21] Barone V., Cossi M. (1998). Quantum calculation of molecular energies and energy gradients in solution by a conductor solvent model. J. Phys. Chem. A.

[22] Besler B.H., Merz K.M., Kollman P.A. (1990). Atomic charges derived from semiempirical methods. J. Comp. Chem..

[23] Singh U.C., Kollman P.A. (1984). An approach to computing electrostatic charges for molecules. J. Comp. Chem..

[24] Frisch M.J., Trucks G.W., Schlegel H.B., Scuseria G.E., Robb M.A., Cheeseman J.R., Montgomery J.A., Vreven T., Kudin K.N., Burant J.C., Millam J.M., Iyengar S.S., Tomasi J., Barone V., Mennucci B., Cossi M., Scalmani G., Rega N., Petersson G.A., Nakatsuji H., Hada M., Ehara M., Toyota K., Fukuda R., Hasegawa J., Ishida M., Nakajima T., Honda Y., Kitao O., Nakai H., Klene M., Li X., Knox J.E., Hratchian H.P., Cross J.B., Bakken V., Adamo C., Jaramillo J., Gomperts R., Stratmann R.E., Yazyev O., Austin A.J., Cammi R., Pomelli C., Ochterski J.W., Ayala P.Y., Morokuma K., Voth G.A., Salvador P., Dannenberg J.J., Zakrzewski V.G., Dapprich S., Daniels A.D., Strain M.C., Farkas O., Malick D.K., Rabuck A.D., Raghavachari K., Foresman J.B., Ortiz J.V., Cui Q., Baboul A. G., Clifford S., Cioslowski J., Stefanov B.B., Liu G., Liashenko A., Piskorz P., Komaromi I., Martin R.L., Fox D.J., Keith T., Al-Laham M.A., Peng C.Y., Nanayakkara A., Challacombe M., Gill P.M.W., Johnson B., Chen W., Wong M.W., Gonzalez C., Pople J.A. (2004). Gaussian 03, Revision D.01.

[25] Brychtova K., Farsa O., Csollei J. (2008). Synthesis of esters of substituted 6-aminohexanoic acid as potential transdermal penetration enhancers. http://www.usc.es/congresos/ecsoc/12/ECSOC12.htm..

